# Perceptions of donor screening—Do I always need to tell the truth?

**DOI:** 10.1111/tme.70034

**Published:** 2025-10-28

**Authors:** Sheila F. O'Brien, Lori Osmond, Mindy Goldman

**Affiliations:** ^1^ Epidemiology and Surveillance Canadian Blood Services Ottawa Canada; ^2^ School of Epidemiology and Public Health University of Ottawa Ottawa Canada; ^3^ Donation and Policy Studies Canadian Blood Services Ottawa Canada; ^4^ Department of Pathology and Laboratory Medicine University of Ottawa Ottawa Canada

**Keywords:** blood donors, compliance, screening

## Abstract

**Background:**

The Donor Health Assessment Questionnaire (DHQ) is fundamental to blood safety. We describe attitudes towards truthfulness among first‐time donors who tested positive for transfusion transmissible infections and those who did not.

**Methods and Materials:**

From 2005 to 2022 donors positive for infectious markers (cases) and demographically matched controls rated their agreement with statements about truthfulness, privacy and the value of the DHQ.

**Results:**

There were 798 (32% participation) cases and 3192 (39% participation) controls. Most said they read questions carefully (93% cases, 96% controls, *p* < 0.01) and answered truthfully (95% cases, 99% controls *p* < 0.01). Fewer thought the questions make the blood safer (79% cases, 80% controls, *p* = 0.39) and some agreed it is OK not to answer questions truthfully if you know your blood is safe (21% cases, 16% controls, *p* < 0.01). Privacy to answer personal questions was generally adequate (88% cases, 91% controls, *p* < 0.01). Attitudes were similar regardless of paper or electronic DHQ format.

**Conclusion:**

Most first time donors believe they answer screening questions truthfully, but some question the safety benefit to recipients and judge whether they need to be truthful. This was true for donors with positive infectious markers as well as their matched infection‐negative controls.

## 1 INTRODUCTION

Pre‐donation screening questions are fundamental to ensuring safety for both donors and recipients. The effectiveness of the Donor History Questionnaire (DHQ) depends on a range of factors, but donor acceptance and willingness to answer truthfully are clearly integral. When donor criteria changes are implemented, the impact is generally evaluated through monitoring of donation infectious disease rates, pre‐ and post‐implementation compliance surveys, and monitoring of deferrals.

In 2000, Willson et al.^1^ completed cognitive interviews with donors, reporting that they interpret sexual and drug questions as asking about safety and may answer ‘no’ to questions about behaviours they have engaged in if they believe they pose no risk to the recipient. In 2010 we reported findings consistent with this from surveys of donors with a history of intravenous drug use not declared at the time of donation.^2^ In 2020 in a study conducted outside of the donation setting, Sandner et al.^3^ reported that donors generally believe they are truthful, although there may be a tendency towards less honesty for sexual risk questions. To our knowledge, there are no reports describing surveillance of perceptions of donor screening.

Up until 2016, donors self‐completed screening questions on paper questionnaires at Canadian Blood Services, with high‐risk questions pertaining to sexual behaviours and injection drug taking asked in a face‐to‐face interview. The oral format of questioning was introduced historically to ensure donors attended to questions, but there is a risk of social desirability bias in which people tend to provide the answer they think the screener is expecting. In fact, in 2005 about a third of Canadian donors felt they were more likely to answer questions correctly if they were self‐completed.^4^ Computer assisted fully self‐completed questionnaires (e‐DHQ) have been proposed to elicit disclosure of risk factors by creating a greater sense of privacy which reduces the tendency to answer in a way that is socially acceptable.^5^ In 2016 we switched to e‐DHQ in which all questions were self‐completed.

Ongoing surveillance of donors' perceptions of screening is important as screening methods evolve. We have interviewed first‐time donors who tested positive for blood transmissible infections as well as demographically matched control donors since 2005. We aimed to describe donors' perceptions of screening questions and their self‐reported truthfulness. A secondary aim was to determine whether donors' sense of privacy when answering questions increased after introduction of e‐DHQ.

## 2 MATERIALS AND METHODS

### 2.1 Pre‐donation Screening

Canadian Blood Services collects blood donations in all provinces in Canada except Quebec. Donors must be at least 17 years of age. Donors are required to read a pamphlet that explains who should not donate blood prior to each donation which is also available on‐line. However, there are no legal repercussions for failing to acknowledge risk behaviour in screening. Donors complete the DHQ before each donation to assess safety of donation for the donor and risks to recipients of infectious disease. Until July, 2016, donors completed a paper DHQ in‐clinic with the personal questions about sex and drug taking asked by a trained screener in a face‐to‐face interview. Thereafter, an electronic DHQ was implemented in which each question was presented and the donor needed to respond before moving to the next question. The e‐DHQ did not use audio and was completed in a private area in‐clinic. The questions were also re‐organised into a more logical format based on the time frame asked about starting with ‘today’ and ending with ‘ever’, and with items asked as individual questions rather than lists.

### 2.2 Donors Studied (Case–Control)

From April 2005 to December 2022 all first‐time donors with a confirmed positive test result for human immunodeficiency virus (HIV), hepatitis B, hepatitis C and Human T‐cell Lymphotropic virus (HTLV) were invited to participate in a telephone interview about risk factors.^6^, ^7^ From April 2005 to March 2015 donors with confirmed positive syphilis results were invited to participate, after which it was suspended but re‐implemented in January 2020. For each case donor who participated, four control donors who had tested negative for all markers matched according to age (±5 years), sex, donation status and geographic region were randomly selected. A standard notification letter was sent to all infection positive case donors informing them of their test results, advising them to seek medical attention and informing them that they were permanently deferred from blood donation. Donors were then sent a letter inviting them to participate in the telephone interview. Trained interviewers carried out telephone interviews using a pre‐established script. For each completed interview with a case donor, control donors were selected and invited to participate in the same way. If a control donor refused to participate or could not be contacted, another control donor was randomly selected among the eligible donors until four control donors had been interviewed per case donor. The donors were asked about demographics as well as to rate their agreement with statements about their experience completing the DHQ such as their sense of privacy, attitudes towards truthfulness, and the value of the questions to blood safety. The donor was asked to select from a five‐point scale between strongly disagree to strongly agree. Question order was randomised.

## 3 ANALYSIS

The percentages of donors selecting each response in the five‐point scale were calculated for each question in cases and controls separately and displayed in a horizontal bar graph. The mean response to each question and 95% confidence interval was calculated for each question. The percentages of donors agreeing or strongly agreeing were also combined for simplified reporting. Univariate ordered logistic regression models were constructed with each question as the dependent variable and age group, sex, case‐control status and pre‐post e‐DHQ as independent variables. Multiple ordered logistic regression models were then constructed with pre‐post e‐DHQ retained as well as any other independent variables that were significant (*p* < 0.05). McFadden *R* ^2^ values were calculated to compare the improvement in the final model fit with the base intercept model. The McFadden *R* ^2^ is a value between 0 and 1 in which a higher value indicates more improvement in the final model.

## 4 RESULTS

The numbers and percentages of cases and controls who participated are shown in Table 1. Of 2478 cases invited, 798 (32%) participated, and of 8217 controls invited, 3192 (39%) participated. Prior to implementing e‐DHQ, there were 637 cases and 2474 controls and after 161 cases and 718 controls who participated, such that about 78% of the interviewed donors had donated prior to implementation of e‐DHQ. The donors interviewed were demographically similar to all first time donors except there were fewer participants in the youngest age group.

**TABLE 1:** Demographic characteristics of cases and controls (2005–2022).

	Case participants (*N* = 798)	Control participants (*N* = 3192)	*p* value
Age^a^			
<30	226 (28.3%)	870 (28.3%)	0.9822
30–39	144 (18.0%)	553 (25.1%)	
40–49	170 (21.3%)	679 (29.2%)	
50–59	208 (26.1%)	775 (34.3%)	
≥60	50 (6.3%)	199 (11.8%)	
Sex			
Male	495 (62.0%)	1980 (62.0%)	1.0000
Female	303 (38.0%)	1212 (38.0%)	
Positive marker			
HBV	297 (37.2%)	1188 (37.2%)	1.0000
HCV	347 (43.5%)	1388 (43.5%)	
HIV	6 (0.8%)	24 (0.8%)	
HTLV	47 (5.9%)	188 (5.9%)	
Syphilis	101 (12.7%)	404 (12.7%)	
Region			
British Columbia	168 (21.1%)	672 (21.1%)	1.0000
Alberta	154 (19.3%)	616 (19.3%)	
Prairies	49 (6.1%)	196 (6.1%)	
Ontario	397 (49.7%)	1588 (49.7%)	
Atlantic	30 (3.8%)	120 (3.8%)	
Donation date			
Before eDHQ	637 (79.8%)	2474 (77.5%)	0.1576
After eDHQ	161 (20.2%)	718 (22.5%)	

^a^ Note that some of the control donors did not answer the question about age.

**FIGURE 1:**
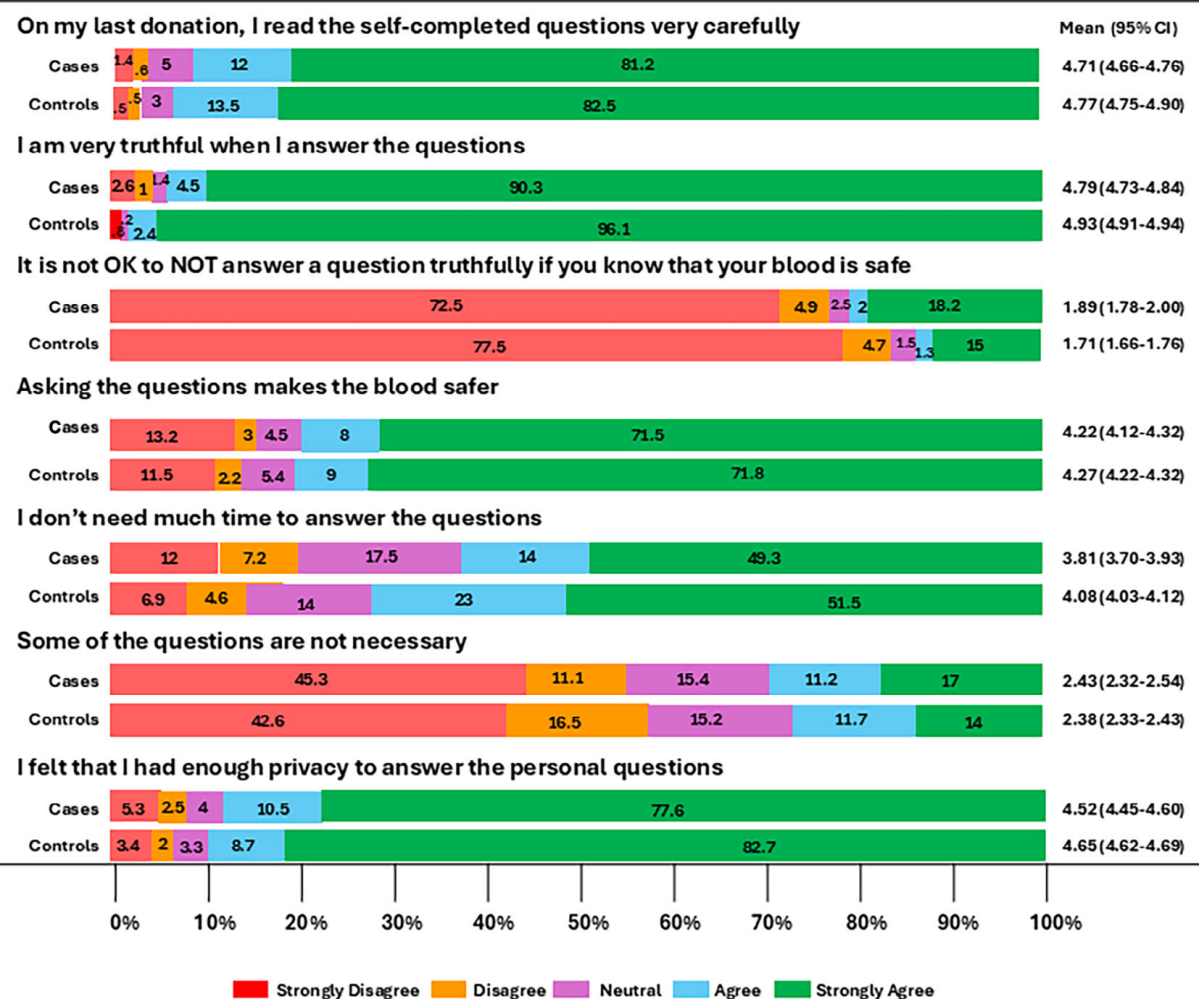
Percentages of donors agreeing with statements about Donor History Questionnaire questions.

**FIGURE A1:**
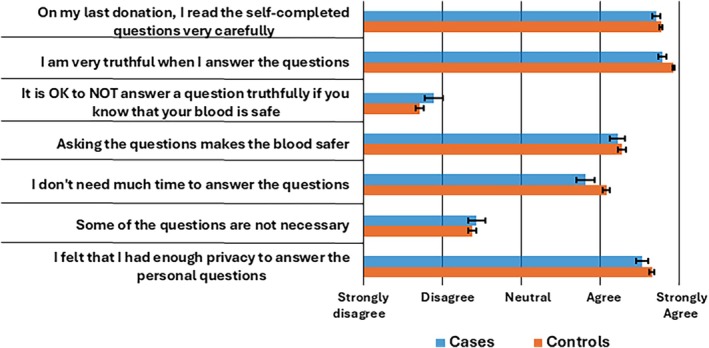
Mean responses of donors' agreement with statements about Donor History Questionnaire questions.

**FIGURE A2:**
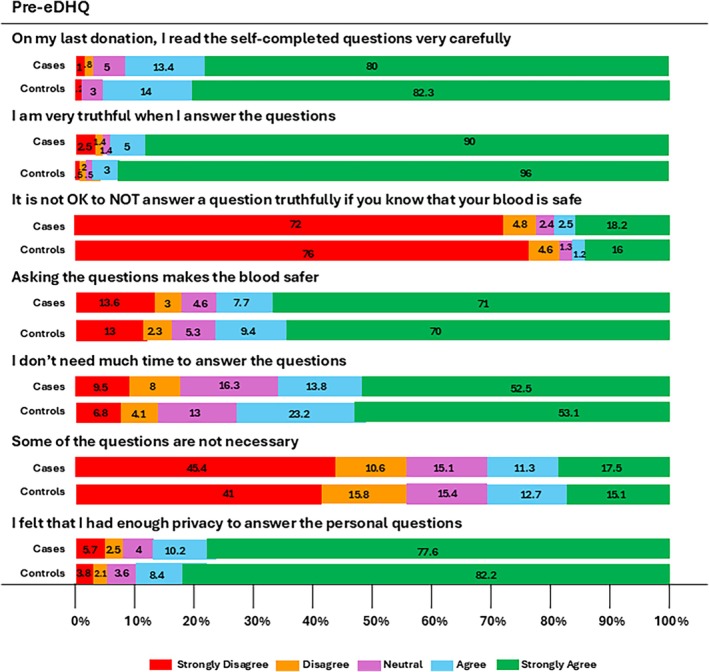
Percentages of donors agreeing with statements about Donor History Questionnaire questions before implementing the electronic Donor History Questionnaire.

**FIGURE A3:**
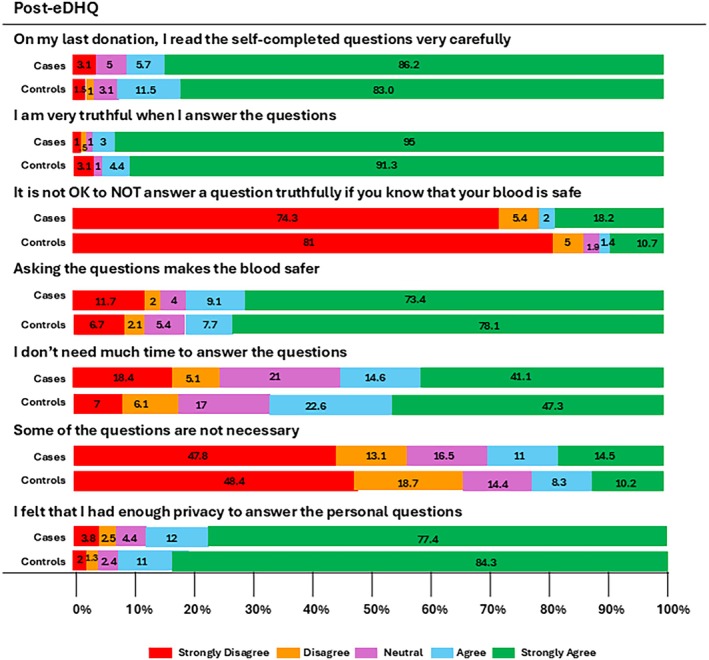
Percentages of donors agreeing with statements about Donor History Questionnaire questions after implementing the electronic Donor History Questionnaire.

The percentages of donors by response for statements about the DHQ are shown in Figure 1. The mean responses of the 5 point scale are shown in Figure A1. Differences between cases and controls were small although sometimes statistically significant. Most donors said they read the self‐completed questions carefully (93% cases, 96% controls, *p* < 0.01) and that they were truthful when answering questions (95% cases, 99% controls *p* < 0.01). Fewer said the questions make the blood safer (79% cases, 80% controls, *p* = 0.39). Cases were somewhat more likely than controls to agree that it is OK not to answer questions truthfully if you know that your blood is safe (21% cases, 16% controls, *p* < 0.01) and somewhat less likely to agree that they had enough privacy to answer the personal questions (88% cases, 91% controls, *p* < 0.01). Fewer donors who thought that it was OK not to answer questions truthfully agreed that asking the questions makes the blood safer (55% vs. 86%, *p* < 0.01). However, donors who thought that it was OK not to answer questions truthfully were similar to those who thought it was not OK for believing that they had been truthful when answering the DHQ (94% vs 99%, *p* > 0.01). The proportions of donors agreeing with statements showed small changes after implementing e‐DHQ (see Figures A2 and A3).

**FIGURE 2:**
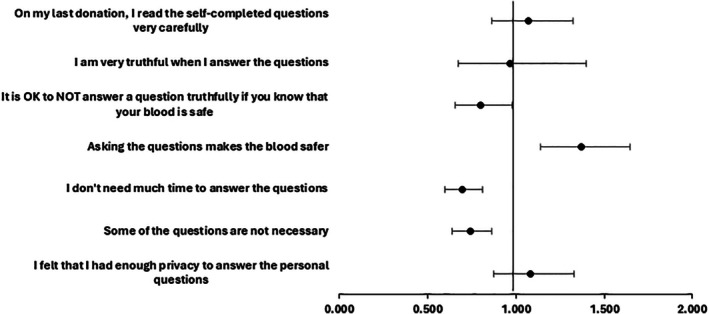
Adjusted odds ratios and 95% confidence intervals for donor agreement with statements about Donor History Questionnaire questions before implementing the electronic Donor History Questionnaire (the reference level) and after implementing. An odds ratio less than one indicates more likely to agree with the statement before implementing eDHQ; an odds ratio greater than one indicates more likely to agree with the statement after implementing eDHQ.

In logistic regression, we included a binary variable for pre‐ versus post‐eDHQ implementation, for which the adjusted odds ratios are shown in Figure 2. The model outputs are shown in Table A1. As shown by the McFadden *R* ^2^ values, the models were little improved over the intercept model. After the implementation of e‐DHQ, donors were more likely to agree that the questions make the blood safer, slightly less likely to say that it is OK not to answer questions truthfully if you know that your blood is safe, but there was no difference in the perception of privacy to answer the personal questions. The logistic regression models also included demographic variables. Age group was not a consistent predictor of agreement with statements except for ‘I don't need much time to answer questions’ in which donors aged 40‐49 (OR 1.4), 50‐59 (OR 1.5), 60+ (OR 2.2) were more likely to agree compared with donors aged 17‐29 (*p* < 0.01). Females were less likely to agree that it is OK not to answer questions truthfully if you know your blood is safe (OR 0.6, *p* < 0.01) and less likely to agree that some of the questions are not necessary (OR 0.7, *p* < 0.01); gender was not a significant predictor of other statements. Cases were less likely than controls to agree that they were truthful (OR 0.4, *p* < 0.01), that they had enough privacy to answer the questions (OR 0.7, *p* < 0.01), that they don't need much time to answer the questions (OR 0.7, *p* < 0.01), but more likely to agree that it is OK not to answer a question truthfully if you know that your blood is safe (OR 1.3, *p* < 0.01).

## 5 DISCUSSION

Our results show that over more than 17 years of monitoring, first time donors generally believe that they are conscientious and truthful in answering screening questions. However, some are less confident of the benefit to blood safety, and make a judgement based on perceived risk to the recipient as to whether or not they need to disclose risk factors when asked directly. These beliefs were consistent across age groups, and there were only small differences between those who tested positive for infectious markers and those who did not. Comparison before and after implementation of eDHQ showed only very small variations in donors' agreement with statements about their DHQ experience. Our report highlights the value that surveillance data can bring to quantifying donor perceptions of screening questions.

Over the time of observation, the screening questions have undergone continual revision and the method of administration of the questionnaire changed. Notably, time deferrals for some percutaneous risks such as tattoo and piercing, and for gay and bisexual men who have sex with men were decreased sequentially.^8^, ^9^ The DHQ underwent a major revision in July, 2016 when the questions were re‐ordered by topic (health history, travel history, lifestyle) and time frame. There was an increase in the number of questions because multi‐item questions were separated into single item questions, and subsequently the number of items asked about has been somewhat reduced. Also, at the same time an e‐DHQ was implemented such that donors could self‐complete the full DHQ. Formerly half of the DHQ was self‐completed and donors answered sexual and injection drug risk questions in a face‐to‐face interview. Our results suggest that none of the above changes were associated with substantive changes in perceived diligence or perceptions that could have a negative impact on safety. At the same time, there is little evidence of improvement. In spite of attaining statistical significance, the differences in percentages of donors agreeing with statements before and after implementing e‐DHQ were small. E‐DHQ has been proposed to improve donors' sense of privacy and to increase reporting of risk behaviours.^5^, ^10^, ^11^ In a study carried out prior to implementing e‐DHQ, we noted that attention to questions was better with e‐DHQ.^12^ Donor agreement that privacy was adequate was unchanged post implementation of e‐DHQ, but there was limited room for improvement since about 90% of donors already agreed pre‐implementation. It is also important to keep in mind that donors using e‐DHQ were first time donors and not able to compare the experience with the former face‐to‐face interviews. Our results may suggest that the sensitive nature of screening questions may cause unease for a minority of donors no matter how they are asked. Other statements rated in our study relating to diligence, truthfulness and the safety benefit of the screening questions may not vary depending on the format of the DHQ because they relate to beliefs about screening itself rather than the process. That such beliefs were independent of age also suggests they are intrinsic to the person.

Surveys addressing donor compliance with screening questions indicate that donors in Canada and other countries are generally compliant with infectious disease risk criteria.^8^ Nearly all of the donors in this surveillance said they read the questions carefully and answered questions truthfully. However, donors appear to make a judgement about their own risk to decide if they need to be truthful when they answer screening questions consistent with an analysis of the ethics of non‐disclosure.^13^ This phenomenon was observed in cognitive interviews with prospective donors in the United States.^1^ In the US study, donors understood that donor screening questions were asking ‘Is my blood safe to donate?’, which framed their answers rather than the literal interpretation of the question. In the UK donors non‐compliant with the male‐to‐male sex deferral frequently said it was not important to declare.^14^ Donors non‐compliant with a lifetime injection drug use deferral said that it was not important to declare because the behaviour was in the past, apparently assessing their blood as being safe for transfusion.^15^ Our study also adds to the literature with a quantitative estimate that about a fifth of the donors did not agree that the questions make the blood safer. This suggests that there could be considerable latitude in interpretation if a donor had deferrable risk. Indeed, compliance surveys suggest that non‐disclosure is correlated with feelings that questions are too personal.^16^

Past compliance surveys suggest that donating with deferrable risk is quite rare.^8^ However, as about a fifth of donors were not convinced that the questions make the blood safer, and more than 15% of donors also thought it OK not to tell the truth if they believed their blood was safe, if more people with deferrable risk present to donate, there could be more non‐compliance. In France donors are deferred if they have had more than one sexual partner in the past 4 months, but compliance is poor, likely because this behaviour is more common and perceived as low risk.^17^ We reported that compliance with temporary deferral for tattoo and piercing was poor, another activity that is common and perceived as low risk.^9^ Donor compliance based on self‐assessment of risk may be particularly problematic when public health messaging differs from donor criteria, such as in the case of pre‐exposure prophylaxis for HIV (PrEP) therapy where usage is increasing.^18^, ^19^, ^20^, ^21^, ^22^ Our results lend insight into how common the perception is that truthfulness in screening is open to interpretation. Our recent report that donor compliance with the new sexual behaviour based criteria was sub‐optimal underscores the operational impact such interpretation may have.^23^ This suggests that donors may need clarification of why questions are being asked.^3^ Further research is needed to better understand why donors may interpret screening questions differently than was intended, and to evaluate mitigation strategies.

Strengths of our data include the long time period of observation, the inclusion of donors who tested positive for transmissible infections with demographically matched controls and randomization of the interview questions to reduce order effects. There are also some important limitations. The use of scripted questions did not permit further exploration of the donor's perspective. The questions asked about the donors' perceptions of screening, but did not assess how well they completed the DHQ. For example, donors rated their agreement with statements about being truthful, but if they didn't understand a DHQ question they may have answered it incorrectly. As control donors' data were anonymized, it was not possible to determine if any tested positive for an infectious marker in a subsequent donation. We did not assess test‐seeking, but differences between cases and controls were small and testing for infectious disease is available free via healthcare providers in Canada. Not all donors invited agreed to participate, hence there may be participation bias. Our surveillance data are observational and could not determine the cause of variation in responses, although variation over the observation period was small. Only first‐time donors were included, and donor attitudes were not followed over time.

In summary, over more than 17 years of surveillance monitoring, most first‐time donors believe that they answer screening questions truthfully, but they are less certain that doing so confers a safety benefit to recipients. First‐time donors appear to make a judgement about their own risk to decide if they need to be truthful when they answer screening questions. This was true for donors who tested positive for infectious markers as well as their matched infection‐negative controls. This has important implications for deferrals of behaviours that donors perceive as low risk.

## AUTHOR CONTRIBUTIONS

SFO developed the study design and oversaw study implementation. SFO and MG conceived the research question. LO carried out analysis of data. SFO prepared the first draft. SFO, LO and MG interpreted the results. All authors contributed to the revision of the manuscript and approved the final submitted version.

## FUNDING INFORMATION

There was no specific funding. This research was funded by Canadian Blood Services through operational funds as part of blood donation surveillance activities and conducted by CBS employees.

## CONFLICT OF INTEREST STATEMENT

The authors have no competing interests.

## PATIENT CONSENT STATEMENT

All donors provided informed consent to participate in this study. For donors under the age of majority parental consent was not requested. Blood donation is accepted from individuals aged 17 and older and does not require parental consent.

## Data Availability

The data that support the findings of this study are available from the corresponding author upon reasonable request.

## APPENDIX A

**TABLE A1:** Ordered logistic regression models.

	Point estimate	95% CI lower	95% CI upper	*p* value	McFadden *R* ^2^
On my last donation, I read the self‐completed questions very carefully					0.0022
Age group (compared with under 30)					
30–39	1.401	1.085	1.809	0.0098	
40–49	1.239	0.981	1.565	0.0726	
50–59	1.149	0.921	1.433	0.2175	
60+	1.243	0.852	1.816	0.2593	
Female (compared with male)					
Cases (compared with controls)					
After eDQH (compared with before)	1.074	0.868	1.327	0.5120	
I am very truthful when I answer the questions					0.0231
Age group (compared with under 30)					
30–39					
40–49					
50–59					
60+					
Female (compared with male)	1.583	1.152	2.174	0.0046	
Cases (compared with controls)	0.377	0.280	0.507	<0.0001	
After eDQH (compared with before)	1.059	0.741	1.513	0.7537	
It is OK to NOT answer a question truthfully if you know that your blood is safe					0.0111
Age group (compared with under 30)					
30–39	0.722	0.575	0.906	0.0050	
40–49	0.940	0.765	1.155	0.5560	
50–59	0.719	0.586	0.884	0.0017	
60+	0.726	0.512	1.031	0.0735	
Female (compared with male)	0.625	0.532	0.735	<0.0001	
Cases (compared with controls)	1.334	1.115	1.595	0.0016	
After eDQH (compared with before)	0.801	0.657	0.977	0.0283	
Asking the questions makes the blood safer					0.0038
Age group (compared with under 30)					
30–39	1.319	1.068	1.629	0.0101	
40–49	1.079	0.891	1.308	0.4358	
50–59	1.328	1.098	1.604	0.0034	
60+	1.341	0.967	1.858	0.0783	
Female (compared with male)					
Cases (compared with controls)					
After eDQH (compared with before)	1.370	1.139	1.648	0.0008	
I don't need much time to answer the questions					0.0091
Age group (compared with under 30)					
30–39	0.996	0.821	1.207	0.9664	
40–49	1.395	1.155	1.684	0.0005	
50–59	1.450	1.214	1.732	<0.0001	
60+	2.219	1.653	2.978	<0.0001	
Female (compared with male)					
Cases (compared with controls)	0.737	0.622	0.873	0.0004	
After eDQH (compared with before)	0.695	0.596	0.810	<0.0001	
Some of the questions are not necessary					0.0052
Age group (compared with under 30)					
30–39	0.836	0.701	0.996	0.0454	
40–49	0.918	0.779	1.083	0.3097	
50–59	0.929	0.793	1.088	0.3604	
60+	0.902	0.693	1.173	0.4407	
Female (compared with male)	0.688	0.609	0.776	<0.0001	
Cases (compared with controls)					
After eDQH (compared with before)	0.743	0.639	0.863	0.0001	
I felt that I had enough privacy to answer the personal questions					0.0067
Age group (compared with under 30)					
30–39	1.268	0.996	1.613	0.0537	
40–49	1.107	0.888	1.380	0.3682	
50–59	1.541	1.230	1.930	0.0002	
60+	2.055	1.347	3.134	0.0008	
Female (compared with male)					
Cases (compared with controls)	0.717	0.592	0.867	0.0006	
After eDQH (compared with before)	1.080	0.877	1.332	0.4686	

*Note*: eDHQ was retained in all models, but other variables were removed if *p* < 0.05.
